# Protein Quality Evaluation of Some Commonly Consumed Bird Egg Varieties Using Amino Acid Scores

**DOI:** 10.1155/2022/6536826

**Published:** 2022-07-12

**Authors:** Eridiong O. Onyenweaku, Levi U. Akah, Hema Kesa, David A. Alawa, Patricia A. Ebai, Ukoha U. Kalu, Ikutal Ajigo, Valentine J. Owan

**Affiliations:** ^1^Department of Human Nutrition & Dietetics, University of Calabar, Calabar, Nigeria; ^2^School of Tourism & Hospitality, University of Johannesburg, Johannesburg, South Africa; ^3^Department of Human Kinetics & Health Education, University of Calabar, Calabar, Nigeria; ^4^Department of Vocational Education, University of Calabar, Calabar, Nigeria; ^5^Department of Home Economics, ENSET, Douala University, Douala, Cameroon; ^6^Department of Zoology & Environmental Biology, University of Calabar, Calabar, Nigeria; ^7^Department of Educational Foundations, University of Calabar, Calabar, Nigeria; ^8^Ultimate Research Network (URN), Calabar, Nigeria

## Abstract

**Objective:**

Food proteins provide amino acids (AAs) and serve as building blocks of all vital organs, muscles, hormones, and biological fluids such as blood. Eggs are known as a good source of protein. This study compared the protein quality of bird eggs (raw and boiled), using their AA scores since some individuals consume raw eggs for various reasons. *Research Methods*. Eggs studied were exotic chicken, local chicken, turkey, quail, and guinea fowl eggs. The eggs were shelled and their contents (boiled and raw) lyophilized. The standard AOAC method (Kjeldahl) was used to determine protein content, while the amino acid composition was measured using an AA analyzer. The total AA scores were calculated based on the whole hen's egg AA profiles. Statistical significance was accepted at *p* < 0.05.

**Results:**

The Guinea fowl egg had the highest total amino acid score (TAAS) of 0.92. The other scores ranged as follows: 0.82 (quail) >0.81 (turkey) >0.75 (exotic chicken), and the lowest score was 0.65 (local chicken). The least scores were phenylalanine: 0.34 (exotic chicken), phenylalanine and serine: 0.36 (local chicken), leucine and aspartic acid: 0.41 (turkey), methionine: 0.31 (quail), and glutamic acid: 0.33 (guinea fowl). Also, guinea fowl egg had the highest total essential amino acid (TEAA) (49.6 g/100 g protein), i.e., % TEAA (55.1%), while exotic chicken egg had the lowest (41.1%), but the highest % NEAA (58.9%).

**Conclusion:**

Guinea fowl eggs had the highest EAA and TAA content. Its consumption should particularly be encouraged for children as this can significantly reduce the risk of protein-energy malnutrition and prevent protein deficiencies.

## 1. Introduction

Diets and nutritious meals are necessary for sustaining good health and avoiding sickness. Recent global increases in malnutrition have prompted many studies on the impact of different cuisines on human health and well-being. Depending on their nutritional composition, certain foods are deemed healthier than others [1]. “The nutrient composition/quality of various foods depends on several factors: species, breeds, cultivars, ecological factors, postharvest handling, preservation, and storage techniques” [1]. In this study, we focused on different eggs and the variation in their protein content.

Foods may be grouped broadly into cereal grains, eggs, fat replacers, fats and oils, fish, fruits, herbs and spices, legumes, meat and poultry, milk and milk products, nuts and seeds, roots and tubers, sweeteners, and vegetables [2]. Each category contains foods that are high in certain key elements. Specifically, animal meals provide more complete proteins than plant diets, including fewer amino acids [3]. Eggs are one of the most common animal protein source foods, and they are frequently consumed in various forms. Eggs are also widely used in the baking and culinary industries for making many baked products and food recipes.

Amino acids (AA) are the building blocks of all critical organs, muscles (particularly heart muscles), hormones, and biological fluids such as blood. A steady supply of high-quality protein is essential for development and other bodily activities since the human body cannot store protein [4]. Hence, the choice of proteins consumed by people of different age groups should be influenced by their amino acid content. Therefore, the protein quality (based on the amino acid composition) of foods is an essential criterion for providing sufficient nutrition and maintaining good health [4].

Protein quality assessment aims to establish a protein's capacity to satisfy maintenance demands and particular needs for development, pregnancy, and lactation [5]. For the majority of individuals, protein intake should equal protein loss. Nonetheless, a positive protein balance is necessary for developing babies and children, during pregnancy and nursing, and during periods of sickness and recuperation [5]. Mothers are encouraged to feed their children with eggs to ensure proper growth and development in many places.

Protein quality may be described as the capacity of a food protein to fulfil the body's metabolic requirements for AA and nitrogen (N). “It is determined by the AA composition and digestibility of the protein and the bioavailability of the individual AA” [6]. The amino acids His, Ile, Leu, Lys, Met, Phe, Thr, Trp, and Val are categorized as “indispensable” (IAA) or “essential” (EAA) since the human body cannot synthesize them; those that the human body can synthesize are classified as “dispensable” or “nonessential” (Ala, Arg, Asn, Asp, Cys, Gln, Glu, Gly, Pro, Ser, and Tyr). In contrast, the endogenous ability to produce some dispensable AA may not always fulfil demand. Theoretically, some de novo synthesis of IAA may occur after urea salvage in the lower abdomen [6]. In addition, “some AA, such as Cys, Tyr, Gly, Arg, Gln, Pro and taurine, are termed “conditionally indispensable” as they become dietary essential only under specific pathological or physiological conditions” [4].

Methods frequently used to evaluate protein quality include “amino acid score (AAS),” “*in vivo* protein digestibility (apparent, corrected or true),” “*in vitro* protein digestibility,” “the protein efficiency ratio (PER),” “estimated PER,” “maximum PER,” “net protein ratio (or retention) (NPR),” “protein rating (PR),” “net protein utilisation (NPU),” “biological value (BV) (apparent, true, relative),” and “the protein digestibility corrected amino acid score (PDCAAS).” “AAS is the ratio of the AA content in 1 g of a target protein to that of a reference protein or requirement” [4].

“The PDCAAS method of protein quality evaluation, the most recently recommended method by the FAO” [4], is based on determining protein content, amino acid profile, and protein digestibility [7]. This study evaluated eggs' protein/amino acid composition, popularly consumed locally and internationally, primarily because eggs are a cheap source of quality protein frequently used to substitute for meat in foods. Previous research also made thought-provoking claims about the medicinal benefits of consuming some fresh (raw) eggs, such as quail eggs. This makes it necessary to look into the contents of these various eggs to ascertain if there are differences in their nutrient content that can account for such claims. Recently published results by Attia et al. [8–10] indicated that the nutritional values of table eggs are affected by genetic and management factors. Furthermore, it was shown that the amino acid concentration of eggs is impacted by poultry types and species, preparation technique, and egg components (whole, white, and yolk) [9]. Due to variations in the amino acid and antioxidant profiles of particular eggs, a review of the scientific literature suggests the potential of enhancing the nutritional value of eggs by modifying their breeds, diets, and management techniques [8, 9]. Nutrient content data of amino acid composition of locally consumed eggs (fresh and boiled) are scarce and need to be updated. Therefore, this study seeks to assess the protein quality of five bird egg varieties and compare their amino acid composition/scores. The eggs studied in this research are exotic chicken, local chicken, turkey, quail, and guinea fowl eggs.

## 2. Materials and Methods

### 2.1. Sample Collection and Identification

Ten fresh egg varieties were bought from Nigerian henhouses in Calabar (Cross River State) and Nsukka (Enugu State). Before being transferred to the laboratory for examination, the varieties were authenticated by Dr Glory Enyenihi of the Animal Science Department, Faculty of Agriculture, University of Calabar, Nigeria [11]. The exotic chicken, turkey, and quail eggs were laid by birds bred in cages. The birds are usually fed with “layer feeds” purchased from the market, and the birds have water available kept in boughs. The local chicken and guinea fowl eggs were laid by free-range birds, who were usually allowed to walk around and feed freely outside (usually on insects, grains, worms, plants, and others) before they returned to roost at night.

### 2.2. Raw Egg Preparation

The contents of the freshly laid eggs were poured into clean, labelled glass beakers after the shells were cleaned and cracked. The contents of the raw eggs were then homogenized and freeze-dried at 40°C for 12 hours, culminating in the sublimation of particles and the desorption of residual water molecules for 10 hours. The homogenized raw egg samples were lyophilized using a freeze-drier (VirTis, Gardener, New York).

### 2.3. Boiled Egg Preparation

The fresh eggs were washed, labelled, and cooked in tap water already boiling at 100 degrees Celsius. There was sufficient tap water to cover the eggs in the pot. After simmering the eggs for 10 minutes, they were removed and allowed to cool in cold tap water at room temperature. Each kind of egg was cooked individually and then peeled after cooling. Before freeze-drying, the eggs were put in clean, labelled beakers and covered with parafilm. The whole (shelled) cooked eggs were mashed and then freeze-dried using a freeze-drier. Using a laboratory miller, all freeze-dried samples were ground into a fine powder with a particle size of around 180 *μ*m (Breville Kitchen Wizz BFP650). The milling process was conducted at a very low temperature. After grinding, 50 g of each sample (raw and boiled freeze-dried samples) was placed in a labelled, sealed sample glass bottle, and refrigerated until analysis [11].

### 2.4. Theory/Calculation: Chemical Analyses

#### 2.4.1. Determination of Crude Protein Content

The Kjeldahl technique was used to determine the protein concentration [12].

Adding extra alkali (concentrated sodium hydroxide) released ammonia gas, then distilled into a boric acid solution to generate the ammonium-borate complex. The ammonia released by the complex is titrated with standardized hydrochloric acid (HCl) to generate a purple-pink endpoint. Based on the milligramme equivalent of the acid employed, the quantity of nitrogen in the sample was estimated. The percentage of nitrogen was computed, and the findings were recorded in triplicate using the formula:

Protein (%) = % total nitrogen *x* appropriate nitrogen conversion factor (6.25).

### 2.5. Determination of Amino Acids by the Amino Acid Analyzer

This involved the hydrolysis of proteins and the separation of free amino acids before quantification. The determination of AA was performed using an amino acid analyzer (Hitachi's High-Speed Amino Acid Analyser LA8080 AminoSAAYA) following the method described by Sun et al. [13]. The analysis time was about 40 mins and the detection limit 2.5 pmol.

The material undergoes a preliminary acid treatment that hydrolyzes proteins and peptides into free amino acids. The resulting free amino acids are separated by ion-exchange chromatography using various pH buffers. The amino acids coming from the column were quantified by combining with ninhydrin to produce a purple-blue colour in proportion to their concentration. Diketohydrindyliden Diketohydrindamin (DYDA), the result of the ninhydrin process, displayed maximal light absorption at 570 nm. The alkaline hydrolysis method was used to quantify tryptophan.

#### 2.5.1. Amino Acid Scoring

The total amino acid scores were determined based on the amino acid profiles of the whole hen's egg [14, 15]:

Amino acid score = amount of amino acid per test protein [mg/g].

Amount of amino: the overall amino acid score for a given egg sample was calculated by adding the individual amino acid scores and getting the average.

### 2.6. Statistical Analysis

The mean and standard error were computed and analyzed using the one-way ANOVA using SPSS version 20 at a significance level of 5%; LSD was used for multiple comparisons. Results were reported as means and standard errors as in Eridiong et al. [11]. Significance was accepted at *p* < 0.05.

## 3. Results

### 3.1. Crude Protein Content of the Eggs (Raw and Boiled)

This is summarised in a column of Table 1. The protein level of the raw egg samples varied substantially (*p* < 0.05), with guinea fowl egg having the most protein content (20.3% 0.15%) and turkey egg having the lowest (11.5% ± 0.44%). Similar trends were detected across the boiled egg samples, with guinea fowl eggs having the most protein content (26.6% ± 0.06%), which was considerably more remarkable than the others (*p* < 0.05), and turkey eggs having the lowest (14.2%, 0.20%).

### 3.2. Amino Acid Composition of the Eggs (Raw and Boiled)

The essential amino acid and nonessential amino acid profiles are presented in Tables 1 and 2.

#### 3.2.1. Essential Amino Acids (EAAs)

All the nine essential amino acids were present in appreciable amounts in raw and boiled samples of the five egg species. Among the EAA, isoleucine and valine had the highest concentrations, while tryptophan had the lowest concentrations. Among both raw and boiled samples, guinea fowl egg had significantly (*p* < 0.05) higher values for all the EAA except for valine which was highest in the exotic chicken egg (6.99 g/100 g protein). Comparing the raw against boiled samples, the concentrations of the EAA were slightly higher in the boiled eggs. Most EAA concentrations varied significantly (*p* < 0.05) among the different egg species, with a few statistically similar values. The raw exotic and local chicken eggs had statistically (*p* > 0.05) similar content of tryptophan (1.45 and 1.51 g/100 g protein, respectively). In contrast, both species in boiled forms also had statistically (*p* > 0.05) similar content of histidine (2.15 and 2.26 g/100 g protein, respectively) and phenylalanine (1.96 and 2.08 g/100 g protein, respectively).

#### 3.2.2. Nonessential Amino Acids (NEAAs)

Nine NEAA were found in appreciable amounts in the five egg species; the NEAA results are presented in Table 2. Aspartic and glutamic acid had the highest concentrations in raw and boiled egg samples, while glycine and cystine had the lowest values. Just as in the essential amino acid results, a similar trend was observed among the nonessential amino acids. Almost all the values were statistically (*p* < 0.05) different within the various groups, with only a few exceptions. The contents of cystine in both raw (2.52 and 2.43 g/100 g protein) and boiled (2.75 and 2.61 g/100 g protein) exotic and local chicken eggs, respectively, were statistically (*p* > 0.05) similar. Similarly, exotic chicken and quail egg had statistically (*p* > 0.05) similar content of alanine (4.14 and 4.10 g/100 g protein, respectively).

#### 3.2.3. Summary of the Amino Acid Concentration

The total EAA (TEAA), total NEAA (TNEAA), TAA, percentage of the essential amino acid (% EAA), and percentage of the nonessential amino acid (% NEAA) are all presented in Table 3. The TEAA ranged between 27.17 and 52.35 g/100 g protein among the raw and boiled samples. In the raw egg samples, guinea fowl egg had the highest TEAA (49.6 g/100 g protein), TAA (90.0 g/100 g protein), and % TEAA (55.1%), while exotic chicken egg had the lowest % TEAA (41.1%) but the highest % NEAA (58.9%). The total amino acid in g/100 g protein (for raw samples) ranged as follows: 90.0 (guinea fowl) >80.9 (quail) >76.2 (exotic) >71.3 (Turkey) >62.1 (local chicken). The TEAA for the raw samples ranged between 27.17 and 49.58 g/100 g protein, with local chicken egg having the lowest while guinea fowl egg had the highest value. The TNEAA for the raw samples ranged between 34.92 g/100 g protein (local chicken egg) and 45.73 g/100 g protein (quail egg).

#### 3.2.4. Amino Acid Scores of the Bird Eggs

Table 4 presents the calculated amino acid scores of the five egg species based on the whole hen's egg profile (FAO/WHO, 1991; Paul & Southgate, 1976). Guinea fowl egg had the highest total amino acid score (TAAS) of 0.92. The other scores ranged as follows: 0.82 (quail) >0.81 (turkey) >0.75 (exotic chicken) and the lowest score was 0.65 (local chicken).The individual scores had values greater than 1 in Cys (1.40) in exotic chicken eggs; Cys (1.35) in local chicken eggs; His (1.39), Trp (1.10), and Cys (1.48) in turkey eggs; and Asp (1.07) and Cys (1.24) in quail egg; in guinea fowl egg, the scores were greater than 1 in His (1.01), Leu (1.11), Lys (1.08), Met (1.10), Phe (1.08), Thr (1.01), Tyr (1.04), and Gly (1.40). The least scores were Phe: 0.34 (exotic chicken), Phe & Ser: 0.36 (local chicken), Leu & Asp: 0.41 (turkey), Met: 0.31 (quail), and Glu: 0.33 (guinea fowl).

## 4. Discussion

All the five egg species in this study contained all the essential amino acids in significant quantities, hence the statement that “egg protein is a complete protein.” Out of the 20 naturally occurring amino acids, 18 were present in the eggs—the 9 EAA and 9 NEAA. Two “neutral” amino acids—glutamine and asparagine (nonessential)—were not found in eggs, and this is confirmed by other similar studies such as Ismail et al. [16] and Lewis et al. [17]. It is also important to note that eggs have no limiting amino acids, unlike incomplete proteins, with one or two limiting amino acids such as methionine and cysteine [18]. A lack of essential amino acids (EAA) like those found in eggs, a well-known animal protein source, may slow development and cause various health issues in humans [8]. It was found that phenylalanine, isoleucine, lysine, isoleucine, valine, and threonine in the complete edible sections of eggs from various egg sources had varied patterns. Eggs' general protein digestibility is 1.0, which should be noted (same as casein, whey protein, and soy).

Guinea fowl eggs had the highest TAA and TEAA content. As a result, it also had the highest AAS making its protein of the highest quality among the five egg species in this study. The values of the TEAA of the eggs were lower than the value of 45.2 g/100 g protein of the egg reference protein [19] except for that of guinea fowl egg. This is similar to the findings of Adeyeye [20], which showed the guinea fowl egg as scoring 0.99 based on the whole hen egg's profile. The ratio of EAA to TAA in the samples was much higher than the 39 per cent deemed sufficient for protein foods for newborns, 26 per cent for children, and 11 per cent for adults [21]. Compared to other animal protein sources, the proportion of EAA/TAA in the samples was favourable: 48.6% in guinea fowl [20], 51.1% in domestic fowl [15], and 50% in whole hen egg [22, 23].

However, most animal proteins are low in cystine (Cys), for example (Cys/TSAA—total sulphur-containing amino acid)%: 35.5% in *Archachatina marginata* [19]; 27.3%–32.8% in female freshwater crab body parts [24]; 23.8%–30.1% in three different Nigerian fishes [25]; 26.0–26.5% in turkey meat [26]; and 44% in domestic fowl [14]. The present Cys/TSAA% results, such as 46.9% (turkey egg) and 56.8% (exotic chicken egg), differ from these literature observations except for that of guinea fowl (20.4%), which was low like that reported by Adeyeye [20]. This means that egg protein has a good cystine content, unlike some animal proteins. Numerous vegetable proteins have much more cystine than methionine (two sulphur-containing amino acids), such as 62.9% in coconut endosperm [27] and 44.5% in entire Bambara groundnut seeds [28]. Cystine positively affects mineral absorption, particularly zinc [29, 30].

The three branched-chain amino acids (BCAA), leucine, isoleucine, and valine, were also present in reasonable amounts, as seen in the amino acid scores. Guinea fowl eggs had significantly higher amounts of all three BCAA, and their AAS were relatively high. The BCAA are essential for muscle protein synthesis and can also be oxidized in the muscles during exercise for energy. A study by Blomstrand et al. [31] showed that an intake of BCAA improved both mental and physical performances during athletic events. It is also important to note that boiling the eggs did not reduce the AA content of the eggs. Instead, an increase in the AA values was generally observed in both the EAA and NEAA. This is similar to Amaechi et al. [32], who reported that processing methods, mainly roasting, increased the AA composition of some flours. The situation is different if the eggs were boiled for more prolonged periods; however, those in this study were boiled following standard procedures for 10 mins.

Furthermore, results from similar studies state that “the difference in protein and AA patterns of various eggs could be attributed to the impact of the diet composition on the level of crude protein and amino acids” [8, 33, 34]. This implies that if the birds consume high protein feeds, their protein content and quality may be enhanced. These findings are consistent with those reported by [9, 16] that regular raw eggs laid by free-range village hens (which were not allowed to roam outside but were allowed to roam in a building or open area) and nutrient-enriched eggs from poultry have different amino acid patterns, with lysine, leucine, isoleucine, and valine having the highest concentrations in the eggs. Limiting EAA for humans (which cannot be generated in sufficient amounts by human cells), phenylalanine, methionine, lysine, leucine, isoleucine, valine, threonine, and tryptophan [35] were all present in the eggs, unlike some other plant-sourced proteins. Furthermore, due to their physiological requirements, histidine, arginine, cysteine, and tyrosine are considered EAA for newborns and developing children [9].

Biochemically, variations in the amino acid patterns of egg proteins may be ascribed to the kind and proportion of protein in the egg white and egg yolks, such as ovalbumin, ovotransferrin, lysozyme, immunoglobulin Y, and ovomucin [9]. More than fifty per cent of the protein in egg albumen is ovalbumin [36]; it is the predominant protein. From the results of this study, cystine (1.40 AAS) was the most significant amino acid in eggs, while (0.34 ASS) was the lowest. In a study by Attia et al. [9], eggs included more significant arginine, serine, cysteine, and isoleucine amino acids than soy protein, beef, casein, wheat flour, and egg white [37].

## 5. Conclusion

This research showed that the five egg species under study had nine essential amino acids in significant amounts and nine nonessential amino acids. Guinea fowl egg had higher amino acid score than the standard reference (hen's egg). In terms of heating, it was observed that boiling the eggs for just 10 mins under standard conditions did not reduce the amino acid content of the eggs; instead, a slight increase was observed in almost all the AA values of the boiled samples. In addition, the results show that not all eggs are the same in protein quality and certain varieties may be better recommended for frequent consumption by children who have higher protein requirements for growth and development. Consumption of less popular varieties such as guinea fowl and quail eggs should also be promoted due to their relatively higher nutritional value.

## Figures and Tables

**Table 1:** Essential amino acid composition of raw and boiled egg varieties.

Egg species	Essential amino acids (g/100 g protein)									
	Protein (%)	His	Ile	Leu	Lys	Asn	Phe	Thr	Trp	Val
*Raw*										
Exotic chicken	15.2 ± 0.07^c^	1.96^d^	4.06^c^	5.57^c^	4.14^d^	1.92^d^	1.74^e^	3.43^d^	1.45^c^	6.99^a^
Local chicken	12.7 ± 0.25^d^	2.06^c^	3.25^e^	6.69^b^	3.18^e^	2.02^c^	1.86^d^	2.48^e^	1.51^c^	4.12^e^
Turkey	11.5 ± 0.44^e^	3.34^a^	4.23^b^	3.43^d^	5.27^b^	3.01^b^	3.88^c^	3.93^c^	1.98^a^	4.43^d^
Quail	15.6 ± 0.10^b^	2.15^c^	3.86^d^	7.12^b^	4.71^c^	0.97^e^	4.08^b^	4.64^b^	1.23^d^	6.38^b^
Guinea fowl	20.3 ± 0.15^a^	2.42^b^	5.38^a^	9.21^a^	6.70^a^	3.52^a^	5.51^a^	5.15^a^	1.70^b^	6.15^c^

*Boiled*										
Exotic chicken	19.3 ± 0.03^b^	2.15^c^	4.54^b^	6.12^d^	4.68^d^	2.03^d^	1.96^e^	3.64^d^	1.68^d^	7.24^a^
Local chicken	18.6 ± 0.38^b^	2.26^c^	3.75^d^	6.84^c^	3.39^e^	2.27^c^	2.08^d^	2.71^e^	1.92^b^	4.89^d^
Turkey	14.2 ± 0.20^d^	3.94^a^	4.61^b^	3.62^e^	5.54^b^	3.25^b^	4.11^c^	3.99^c^	2.07^a^	4.66^e^
Quail	16.1 ± 0.15^c^	2.98^b^	3.99^c^	7.40^b^	5.08^c^	1.16^e^	4.29^b^	4.68^b^	1.46^c^	6.92^b^
Guinea fowl	26.6 ± 0.06^a^	3.07^b^	5.92^a^	9.39^a^	6.94^a^	3.96^a^	6.45^a^	5.29^a^	1.95^b^	6.38^c^

Values are expressed as mean ± SEM, *n* = 3; note: all the SEMs were less than 0.005; hence they were rounded off; values in the same column with different superscripts are significantly different (*p* < 0.05).

**Table 2:** Nonessential amino acid composition of raw and boiled egg varieties.

Egg species	Nonessential amino acids (g/100 g protein)								
	Tyr	Ala	Arg	Asp	Cys	Glu	Gly	Pro	Ser
*Raw*									
Exotic chicken	2.19^e^	4.14^b^	3.62^d^	8.85^c^	2.52^b^	11.23^a^	2.50^c^	2.66^d^	7.10^a^
Local chicken	2.37^d^	2.26^d^	3.83^c^	7.88^d^	2.43^b^	9.00^b^	1.51^e^	2.82^c^	2.82^e^
Turkey	3.07^b^	2.95^c^	4.85^b^	4.39^e^	2.66^a^	7.44^d^	1.92^d^	3.63^b^	6.87^b^
Quail	2.76^c^	4.10^b^	4.94^b^	11.45^a^	2.23^c^	8.40^c^	2.75^b^	2.38^e^	6.72^c^
Guinea fowl	4.16^a^	4.91^a^	6.04^a^	9.10^b^	0.90^d^	3.96^e^	4.20^a^	3.76^a^	3.40^d^

*Boiled*									
Exotic chicken	2.38^e^	4.52^b^	3.91^d^	9.14^c^	2.75^b^	11.50^a^	2.73^c^	2.77^c^	7.70^a^
Local chicken	2.76^d^	2.71^d^	3.97^d^	8.24^d^	2.61^b^	9.85^b^	1.82^e^	2.98^b^	3.17^e^
Turkey	3.48^b^	2.34^e^	5.12^c^	4.76^e^	2.97^a^	7.46^d^	1.96^d^	3.91^a^	7.50^b^
Quail	2.94^c^	4.44^c^	5.23^b^	11.96^a^	2.38^c^	8.74^c^	2.92^b^	2.56^d^	6.98^c^
Guinea fowl	4.45^a^	5.05^a^	6.29^a^	9.35^b^	1.12^d^	4.18^e^	4.46^a^	3.94^a^	3.55^d^

Values are expressed as mean ± SEM, *n* = 3 at *p* < 0.05; note: all the SEMs were less than 0.005, hence rounded off as zero; values in the same column with different superscripts are significantly different (*p* < 0.05).

**Table 3:** Summary of amino acid concentrations.

Egg species	TEAA^*∗*^	TNEAA^*∗*^	TAA^*∗*^	% EAA	% NEAA
*Raw*					
Exotic chicken	31.26	44.81	76.07	41.10	58.90
Local chicken	27.17	34.92	62.09	43.80	56.20
Turkey	33.25	37.78	71.03	46.60	53.40
Quail	35.14	45.73	80.87	43.40	56.60
Guinea fowl	49.58	40.43	90.01	55.10	44.90

*Boiled*					
Exotic chicken	34.04	47.40	81.44	41.80	58.20
Local chicken	30.11	38.11	68.22	44.10	55.90
Turkey	35.79	39.50	75.29	47.50	52.50
Quail	37.96	48.15	86.11	44.10	55.90
Guinea fowl	52.35	42.39	94.74	55.20	44.80

^*∗*^Total values are expressed in g/100 g protein. TEAA means total essential amino acid, TNEAA means total nonessential amino acid, TAA means total amino acid, % EAA means percentage essential amino acid and % NEAA means percentage nonessential amino acid.

**Table 4:** Amino acid scores (AAS) of bird egg varieties.

Amino acid	Exotic chicken	Local chicken	Turkey	Quail	Guinea fowl
Histidine	0.82	0.86	1.39	0.90	1.01
Iso leucine	0.72	0.58	0.75	0.69	0.96
Leucine	0.67	0.81	0.41	0.86	1.11
Lysine	0.68	0.51	0.85	0.76	1.08
Methionine	0.60	0.63	0.94	0.61	1.10
Phenylalanine	0.34	0.36	0.76	0.80	1.08
Threonine	0.62	0.49	0.77	0.91	1.01
Tryptophan	0.81	0.84	1.10	0.68	0.89
Valine	0.93	0.56	0.59	0.85	0.82
Tyrosine	0.55	0.59	0.77	0.69	1.04
Alanine	0.77	0.42	0.55	0.76	0.91
Arginine	0.59	0.63	0.79	0.81	0.99
Aspartic acid	0.82	0.74	0.41	1.07	0.85
Cystine	1.40	1.35	1.48	1.24	0.50
Glutamic acid	0.85	0.75	0.62	0.70	0.33
Glycine	0.83	0.50	0.64	0.92	1.40
Proline	0.70	0.74	0.96	0.62	0.99
Serine	0.90	0.36	0.87	0.85	0.43
Total AAS	**0.75**	**0.65**	**0.81**	**0.82**	**0.92**

Total AAS is the overall amino acid score of a particular sample.

## Data Availability

Data are available on request.
